# 3-Phenylcoumarins as Inhibitors of HIV-1 Replication

**DOI:** 10.3390/molecules17089245

**Published:** 2012-08-02

**Authors:** Dionisio Olmedo, Rocío Sancho, Luis M. Bedoya, José L. López-Pérez, Esther del Olmo, Eduardo Muñoz, José Alcamí, Mahabir P. Gupta, Arturo San Feliciano

**Affiliations:** 1 Department of Pharmaceutical Chemistry, Faculty of Pharmacy, University of Salamanca, CIETUS-IBSAL, 37007 Salamanca, Spain; Email: dolmedo_agudo@hotmail.com (D.O.); olmo@usal.es (E.D.O.); asf@usal.es (A.S.F.); 2 CIFLORPAN, Center for Pharmacognostic Research on Panamanian Flora, College of Pharmacy, University of Panama, Box 0824-00172, Panama, Republic of Panama; Email: mahabirpgupta@gmail.com; 3 Department of Cellular Biology, Physiology and Immunology, University of Córdoba, Institute Maimonides for Biomedical Research (IMIBIC). Avda de Menendez Pidal s/n, 14004 Córdoba, Spain; Email: Rocio.Sancho@cancer.org.uk (R.S.); fi1muble@uco.es (E.M.); 4 National Microbiology Centre Institute Carlos III, Crt. Majadahonda a Pozuelo, 28220 Majadahonda, Madrid, Spain; Email: lmbedoya@isciii.es (L.M.B.); ppalcami@isciii.es (J.A.)

**Keywords:** 3-phenylcoumarins, AIDS, Tat protein, NF-*κ*B inhibition, anti-HIV activity

## Abstract

We have synthesized fourteen 3-phenylcoumarin derivatives and evaluated their anti-HIV activity. Antiviral activity was assessed on MT-2 cells infected with viral clones carrying the luciferase gene as reporter. Inhibition of HIV transcription and Tat function were tested on cells stably transfected with the HIV-LTR and Tat protein. Six compounds displayed NF-*κ*B inhibition, four resulted Tat antagonists and three of them showed both activities. Three compounds inhibited HIV replication with IC_50_ values < 25 µM. The antiviral effect of the 4-hydroxycoumarin derivative **19** correlates with its specific inhibition of Tat functions, while compound **8**, 3-(2-chlorophenyl)coumarin, seems to act through a mechanism unrelated to the molecular targets considered in this research.

## 1. Introduction

Human immunodeficiency virus (HIV) infection is the cause of acquired immunodeficiency syndrome (AIDS) [1], one of the leading causes of morbidity and mortality in the World [2]. Although Highly Active Antiretroviral Therapy (HAART) has resulted highly effective in suppressing HIV load and in decreasing mortality of AIDS patients, current treatments do not eradicate the virus from the infected host. After HIV entry into the host cell, viral RNA is transcribed to proviral DNA, transported to cell nucleus and stably integrated into the host genome. At this point, depending on several cellular and viral factors, provirus can be actively transcribed to viral RNAs or remain in a latent state, integrated in the genome of CD4+ cells, constituting viral reservoirs [3], not easily reached by HAART [4]. Thus, long-term treatments are needed to prevent viral reactivation after therapy discontinuation, leading to drug adverse effects and emergence of viral resistances. Novel targets for anti-HIV drugs are necessary, and viral transcription seems to be particularly relevant in this context. Among viral and cellular factors involved in HIV transcription, the NF-*κ*B/Rel family of cellular transcription factors and the viral protein Tat, are the most important factors in Long Terminal Repeat (LTR)-induced HIV transcription [3]. Therefore, inhibition of the activity of these critical proteins should result in an effective blocking of viral replication. Failures in efforts to develop an effective vaccine against HIV-1 infection [5], have also emphasized the importance of antiretroviral therapy for treating AIDS patients. Therefore, medicinal chemists are interested in the development of novel anti-HIV agents that might be particularly effective in controlling the HIV strains resistant to the current drugs [6].

The viral cycle of the HIV can be divided into early and late stages. Early stages comprise several steps, from viral attachment on the cell surface to integration in the host genome. Late stages include the processes of HIV mRNA synthesis, protein expression and morphogenesis. Once integrated, HIV can remain in a latent state in resting lymphocytes or undergo active replication. Transition from latency to HIV expression occurs mainly when cells are activated and requires the concerted action of cellular transcription factors and regulatory HIV proteins [7]. Among the transcription factors involved in LTR transactivation, the HIV proximal enhancer contains three binding sites for SP1 transcription factor and two binding sites for NF-*κ*B. The NF-*κ*B/Rel family of transcription factors represents a major inducible regulatory element involved in HIV transcription. Located downstream of the basal promoter TAR sequence is the RNA target for the viral protein Tat, which acts in concert with other cellular factors to generate full-length RNA transcripts [8]. Furthermore, NF-*κ*B and Tat cooperate in driving HIV replication from the state of latency [9]. Therefore, inhibition of the activity of these critical proteins should result in an effective blocking of viral replication [10,11].

**Figure 1:**
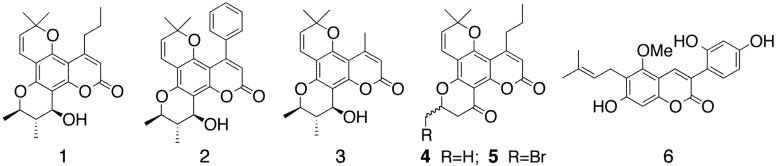
Natural and synthetic anti-VIH coumarins.

Coumarins are well-known natural products displaying a broad range of biological activities [12,13]. More recently, other activities have been described for these compounds such as antidepressant [14], vasorelaxant and platelet-aggregation inhibition [15], enzymatic inhibition of MAO [16,17,18], antioxidant activity [19], and proapoptotic activity [20,21]. The discovery, structural modification and structure-activity relationships studies of coumarins as anti-HIV agents, have been the object of several updates and reviews [22,23,24]. It has been reported that natural tetracyclic dipyranocoumarins with *n*-propyl (calanolides, **1**) [25], or phenyl (inophyllums, **2**) [26], or methyl (cordatolides, **3**) groups attached to position C-4 of the coumarin skeleton (Figure 1) and other related synthetic derivatives, display substantial anti-HIV activity, through several mechanisms, including blockade of viral entry, inhibition of reverse transcriptase or interference with viral integration [27,28]. Some phenylcoumarins, have also been proposed as suppressors of HIV transcription, but the mechanism of action has not been fully characterized [29].

Although optically active (+)-calanolide A (**1**) has been successfully synthesized in the laboratory through different procedures [30], its related 11-demethyl-12-oxocalanolide A (**4**), with less chiral centers, and used as a racemate, has shown comparable *in vitro* anti-HIV activity and even a better selectivity than (+)-calanolide A. Furthermore, the bromoderivative **5** has demonstrated much higher potency and selectivity [29]. According to these findings, it seemed clear that, structural changes at least, at positions C3-C4 and at the prenyl parts of the molecule, could be introduced while retaining or even enhancing the anti-HIV activity. Indeed, natural 3-phenylcoumarins have shown different types of biological properties. Among them, the prenyl derivative licopyranocoumarin (**6**), isolated from licorice, specifically suppresses the tetradecanoyl phorbol acetate (TPA)-induced HIV promoter [29]. In addition, several synthetic 3-phenylcoumarins have demonstrated their inhibitory activity on the HIV-1 protease at the μM range [31].

In a previous paper we reported on the HIV replication inhibition by a series of natural 4-phenylcumarins (neoflavones) isolated from *Marila pluricostata* [32], followed by another report describing the NF-*κ*B transcription pathway as the target for mesuol, one representative of those tested coumarins [33]. They were structurally related to the inophyllum series of coumarins, although without cyclisation of the prenyl groups across the 5- and/or 7-hydroxyl groups [34]. Furthermore, these compounds were tested *in vitro* against sensitive and multi-drug-resistant strains of *Mycobacterium tuberculosis (MTB)* with positive results, which will be published shortly. These relevant facts induced us to continue our research focused on the synthesis of new neoflavones and 3-phenylcoumarin derivatives. We aimed to enlarge the structural diversity to be evaluated and to obtain rather simple and readily accessible compounds, which could display enhanced anti-HIV and anti-TB activities. In this paper we report on the synthesis and the results of anti-HIV evaluation of fourteen structurally diverse 3-phenylcoumarin derivatives.

## 2. Results and Discussion

**Scheme 1:**
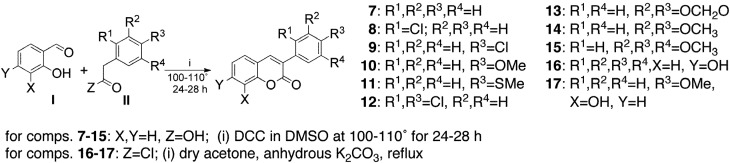
The synthesis of the 3-phenylcoumarins **7–17**.

3-Phenylcoumarins have been synthesized by a variety of methods [35,36]. In our hands, direct condensation of 2-hydroxybenzaldehyde (salicylic aldehyde, SA) derivatives with substituted phenylacetic acids (PAAs) by the original Perkin’s procedure, did not give good yields of the desired products. Several modifications of the procedure were further applied, with results mainly dependent on the type of substituent at the ring of the aldehyde substrate. A simple and convenient procedure, which we have now used to prepare the phenylcoumarins **7–15** is based on the reaction between SA derivatives (**I**) and ring-substituted PAAs (**II**), using dicyclohexylcarbodiimide (DCC) in DMSO (Scheme 1). With the objective of optimizing the reaction conditions, different reaction temperatures were tested, and we found that the best results were attained when heating the mixture at 100–110 °C for 24–28 h [37]. Lower temperatures proved to be less effective, while higher temperatures led to very complex mixtures in the crude reaction products. However, under the best conditions, important differences in yields, mainly determined by the type of substituent attached to the PAA ring, were observed. As an example, condensation of 4-chloroPAA with SA led to the expected coumarin **9** in 60% yield, whereas the *ortho* isomer 2-chloroPAA led to only a 30% yield of coumarin **8**. 3-Phenylcoumarins **16** and **17** were prepared by a slightly different procedure (Scheme 1). To obtain both compounds in good yield, instead of the free carboxylic acids, PAA and 4-methoxy PAA, the corresponding PPA chlorides, were heated to reflux with resorcinol and catechol derivatives, respectively. The reaction was done in dry acetone and in the presence of anhydrous potassium carbonate [38]. Under such conditions these compounds were obtained in yields of 92% (**16**) and 90% (**17**) (Scheme 1).

**Scheme 2:**
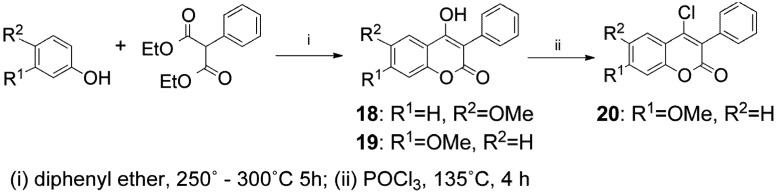
The synthesis of 3-phenylcoumarins **18–20**.

Methods reported in the literature for the preparation of 3-phenylcoumarins **18–20** involve multistep procedures with low yields [39]. However, heating of diethyl 2-phenylmalonate with the appropriate substituted phenols in diphenyl ether, above 250 °C, can afford compounds **18** and **19 **with better yields [40] (Scheme 2). Subsequent reaction of compound **19**, with excess of POCl_3_, leads to 4-chlorocoumarin **20**in high yield.

As it can be seen, the 3-phenyl group is present in all the coumarins tested. Other substituents differ in nature and/or location on the coumarin system, thus configuring a series of close yet enough varied structural arrangements, despite the small number of coumarins involved. Furthermore, these compounds contain common and less common natural-like electron-donating groups (hydroxyl, methoxyl, methylenedioxy, trimethoxyphenyl, methylsulfanyl), and the most typical electron-withdrawing chlorine substituent. A complete description of the chemical procedures used for the synthesis of 3-phenylcoumarins and characterization data for the compounds included in this study are given in the experimental part.

In the characterization of anti-HIV activity, the effect of these coumarins on Tat and NF-*κ*B functions has been evaluated in a similar manner as we described in a previous publication [41]. We have used two transfected 5.1 cell lines, in which we measured NF-*κ*B activation induced by TNFα [42] and HeLa-Tat-Luc cells, in which the HIV-1 LTR is directly activated by the HIV-1 Tat protein. Most of the compounds were submitted for further evaluation through a HeLa-Tet-ON assay in order to determine the selectivity of their action [41]. To avoid missing compounds acting through another mechanism, all substances were also evaluated using a recombinant virus (RV) assay. In this system, a luciferase reporter gene was cloned in full-length infectious DNA. As a result, the measured luciferase activity is therefore proportional to viral replication [43].

The results of anti-HIV evaluation of coumarins **7–20** are shown in Table 1. Data for AZT and mesuol [33], a prenyl derivative of 4-phenylcoumarin, have been included for comparison purposes. In a preliminary analysis of the results, it can be observed that coumarins **10**, **11**, **13**, **15**, **16** and **17** showed a fair inhibitory activity in the NF-*κ*B test at 50 μM, and this behaviour was also confirmed for two of them, the phenylcoumarins **11** and **17**, at 25 μM. Regarding the HeLa-Tat-Luc and Hela-Tet-ON assays, coumarins **10**, **11** and **17** also showed a potent though unspecific anti-Tat activity, while compound **19** showed a modest, but specific inhibition of this target. Unfortunately, coumarins that revealed higher inhibitory potencies in both assays (coumarins **17**, **11** and **10**) displayed unspecific modes of action through the HeLa-Tet-ON evaluation.

**Table 1:** Results of *in vitro* anti HIV-1 evaluation of 3-phenylcoumarins.

Compound	Antiviral activity						
	5.1 + TNFα		Hela-Tat-luc		HeLa-Tet-ON	RV (VIH)	Toxicity (%)
	25 µM	50 µM	25 µM	50 µM	50 µM	IC_50_, µM	CC_50_
**7**	nt	21.7 ± 3	26.8 ± 2.9	33.7 ± 1.0	S	130 ± 11.4	>50
**8**	nt	−28.7 ± 4	nt	17.6 ± 2.1	S	20.7 ± 1.9	>50
**9**	nt	33.0 ± 2.33	nt	−4.9 ± 0.8	nt	>195	>50
**10**	nt	61.2 ± 2.4	73.5 ± 6.7	66.6 ± 3.4	U	73 ± 8.6	>50
**11**	84.3 ± 3	81.6 ± 5.6	77.5 ± 8.5	80.1 ± 5.6	U	18.2 ± 0.2	>50
**12**	nt	−40.9 ± 2.3	nt	14.1 ± 0.6	S	>172	>50
**13**	nt	68.5 ± 3.4	32.8 ± 4.3	39.5 ± 2.3	S	138 ± 22,3	16.7 ± 3.4
**14**	nt	−23.0 ± 2.9	nt	−25.1 ± 5.3	nt	>177	>50
**15**	nt	49.7 ± 7.0	47.6 ± 6.2	35.1 ± 3.9	U	62.7 ± 13.1	42.8 ± 16.2
**16**	nt	59.0 ± 8.2	nt	17.2 ± 0.5	S	81.4 ± 14.0	>50
**17**	82.2 ± 3.1	87.8 ± 9.8	85.7 ± 3.4	88.3 ± 5.5	U	84.6 ± 12.1	>50
**18**	nt	−3.5 ± 0.9	nt	22.8 ± 3.4	S	186 ± 10.8	>50
**19**	−34.26 ± 5	−4.7 ± 0.3	48.9 ± 3.4	55.7 ± 4.9	S	23.8 ± 1.7	>50
**20**	nt	−21.2 ± 7.0	nt	−64.4 ± 10	nt	>209	18.4 ± 8.2
**Mesuol**	71.0 ± 4.8	77.9 ± 4.9	nt	71.3 ± 8.9	S	2.5	>4 µM
**AZT**	nt	nt	nt	nt	nt	0.01	>1 µM

S: specific; U: unspecific modes of action; compounds considered active in each essay are boldfaced.

More interestingly, coumarins **13**, and **16** (3-phenylumbelliferone) resulted as specific inhibitors of the NF-*κ*B function. Compound **15**, showed a lower potency and was unspecific. It must be noted that simple structural differences within this series of 3-phenylcoumarins determine substantial changes in activity and selectivity. As an example, we could compare the NF-*κ*B inhibitor 3-phenylumbelliferone (**16**) and the specific Tat inhibitor **19**, also a 3-phenylumbelliferone-related coumarin. The drastic difference in bioactivity for these compounds should be due either to the presence of the hydroxyl group at C-4, or to the methylation of the hydroxyl group at C-7 or to both modifications. However, regarding the comparison of compound **13** with **16**, it is worth noting that the introduction of a methylenedioxy fragment on the 3-phenyl substituent, not only retains but also enhances the NF-*κ*B inhibitory activity. It should be also noted that compound **13** lacks the 7-hydroxyl group, which could play an important role in the inhibitory activity.

Concerning the results of the antiviral RV assay, three 3-phenylcoumarins displayed IC_50_ values < 25 μM and can be considered as potent replication inhibitors. As it could be expected, the sulfanylphenylcoumarin **11**, one of the most potent dual-target inhibitors, resulted also the most potent inhibitor of HIV replication. Nevertheless, the two other potent dual inhibitors, methoxyphenyl derivatives **10** and **17**, were 4-5-fold lesser potent. None of them was toxic at the concentration used in this study. Looking at the corresponding NF-*κ*B and Tat inhibition values, the second more potent replication inhibitor, the *o*-chlorophenylcoumarin **8**, with a light effect on Tat function only, seems to act, most probably, through another mechanism. On the other hand, the 4-hydroxy derivative **19**, appears as the most interesting compound of this 3-phenylcoumarin series, because of its antiviral potency correlates well with its specific Tat inhibitory activity.

The synthetic phenylcoumarins included in this research, while being more potent than mesuol against both NF-*κ*B and Tat molecular targets, resulted around one-order of magnitude less effective in the RV assay. These facts would suggest that the presence of prenyl and/or prenacyl substituents attached to the aromatic ring of the coumarin nucleus, that are common in natural coumarins, could be a structural factor reinforcing the antiviral activity.

## 3. Experimental

### 3.1. Material and Reagents

All the reagents for synthesis were commercially available and either used without further purification or purified by standard methods prior to use. Reaction progress was monitored using thin-layer chromatography carried out on precoated Merck silica gel Kiesegel 60 F_254_ plates and the spots were detected under UV light (254 nm). Melting points were determined on a Büchi 510-K melting point apparatus and are uncorrected. IR spectra were recorded (KBr 1%) in a Nicolet Impact 410 spectrophotometer. ^1^H, ^13^C-NMR, COSY, HMQC and HMBC were recorded on Bruker AC 200 (200 MHz) and Bruker DRX 400 (400 MHz) instruments. Chemical shifts (δ) are expressed in parts per million (ppm) relative to the residual solvent peak: CDCl_3_ 7.26 ppm/77.0 ppm and MeOD 3.35 ppm/49.0 ppm and coupling constants are reported in Hertz (Hz). For EIMS and HRFABMS analysis, a VG-TS250 mass spectrometer (70 eV) was used. Elemental analyses were performed with a LECO CHNS-932 and were within ±0.4% of the theoretical values.

### 3.2. General Procedure for the Synthesis of Compounds **7–20**

A solution of 2-hydroxybenzaldehyde (1.6 mmol), the appropriate phenylacetic acid (2.0 mmol) and DCC (2.5 mmol) in dimethyl sulphoxide (5.0 mL) was heated in an oil-bath at 100–110 °C for 24–48 h. Cold water (20 mL) and acetic acid (3.0 mL) were added to the reaction mixture; after keeping it at room temperature for 4 h, it was extracted with ether; dicyclohexylurea, which separated at the interface, was filtered and ether layer extracted with aqueous sodium bicarbonate solution (5%, 50 mL). The ether layer was separated and magnetically stirred for 1 h with sodium metabisulphite solution (5%, 3 × 50 mL) to remove the unreacted 2-hydroxybenzaldehyde. The ether layer was separated, washed with water, the solvent distilled off and the residue kept *in vacuo* over phosphorus pentoxide for 24 h. The dry residue on recrystallization from ethyl acetate-methanol afforded the corresponding 3-phenylcoumarins **7–15** as colourless solids.

*3-Phenyl-2H-chromen-2-one* (**7**) was prepared from phenylacetic acid as described in the general procedure; yield 45%; mp 139–141 °C (MeOH). The spectral data (IR, ^1^H-NMR and ^13^C-NMR) were comparable with the data reported in reference [44]. MS (EI) *m/z* 222 (M^+^, 51).

*3-(2-Chlorophenyl)-2H-chromen-2-one* (**8**) was prepared from 2-chlorophenylacetic acid according to the general procedure; yield 30%; mp 186–188 °C (MeOH); IR 3042, 1717, 1710, 1685, 1604, 1489; ^1^H-NMR (CDCl_3_) δ 7.76 (s, 1H), 7.62 (bs, 1H), 7.52 (bs, 1H), 7.42 (bs, 1H), 7.35 (m, 1H), 7.35 (m, 1H), 7.35 (m, 1H), 7.35 (m, 1H), 7.35 (m, 1H); ^13^C-NMR (CDCl_3_) δ 116.7, 119.0, 124.6, 126.9, 127.1, 128.1, 129.9, 130.1, 131.4, 131.9, 133.6, 133.8, 142.7, 154.0, 159.8. MS (EI) *m/z* 256 (M^+^, 42).

*3-(4-Chlorophenyl)-2H-chromen-2-one* (**9**) was prepared from 4-chlorophenylacetic acid using the general procedure described before; 60% yield; mp 190–192 °C (MeOH). The spectral data (IR, ^1^H-NMR and ^13^C-NMR) were quite comparable with the data reported in reference [45]. MS (EI) *m/z* 256 (M^+^, 55).

*3-(4-Methoxyphenyl)-2H-chromen-2-one* (**10**) was prepared from 4-methoxyphenylacetic acid acording to general procedure; yield 55%; mp 141–143 °C (MeOH); IR, ^1^H-NMR and ^13^C-NMR (CDCl_3_) as described in reference [45]. MS (EI) *m/z* 252 (M^+^, 95).

*3-(4-Methylsulfanylphenyl)-2H-chromen-2-one* (**11**) was prepared from 4-methylsulfanylphenylacetic acid according to general procedure; yield 45%; mp 157–159 °C (MeOH); IR 3045, 2918, 1713 ,1686, 1607, 1494; ^1^H-NMR (CDCl_3_) (400 MHz) δ 7.80 (s, 1H), 7.66 (d, *J* = 8.3 Hz, 2H), 7.52 (d, *J* = 8.3 Hz, 1H), 7.50 (dt, *J* = 1.8, 8.3 Hz, 1H), 7.33 (br d, *J* = 8.3 Hz, 1H), 7.31 (d, *J* = 8.3 Hz, 2H), 7.30 (br t, *J* = 8.3, 1.8 Hz, 1H), 2.5 (s, 3H); ^13^C-NMR (CDCl_3_) δ 116.4, 119.7, 124.5, 126.1, 126.1, 127.7, 127.8, 128.8, 128.8, 131.1, 131.3, 139.1, 139.9, 15.5, 15.5, 153.4, 160.6. MS (EI) *m/z* 268 (M^+^, 100).

*3-(2,4-Dichlorophenyl)-2H-chromen-2-one* (**12**) was prepared from 2,4-dichlorophenylacetic using the general procedure; 38% yield; mp 226–228 °C (MeOH); IR 1718, 1701, 1605; ^1^H-NMR (CDCl_3_) δ 7.75 (s, 1H), 7.57 (m, 2H), 7.52 (dd, *J* = 2.0, 8.0 Hz, 1H), 7.51 (m, 1H), 7.41 (br d, *J* = 8.0 Hz, 1H), 7.36 (m, 1H), 7.35 (s, 1H); ^13^C-NMR (CDCl_3_) δ 116.8, 118.9, 124.7, 126.0, 127.3, 128.5, 129.8, 132.2, 132.2, 134.5, 135.4, 143.0, 154.0, 159.7. MS (EI) *m/z* 290 (M^+^, 6).

*3-(Benzo[d][1,3]dioxol-5-yl)-2H-chromen-2-one* (**13**) was prepared from 2-(benzo[*d*][1,3]dioxol-5-yl)acetic acid using the general procedure; 32% yield; mp 168–170 °C (MeOH); IR 3,068, 1,715, 1,609, 1,604; ^1^H-NMR and ^13^C-NMR (CDCl_3_) as described in reference [46]. MS (EI) *m/z* 266 (M^+^, 100).

*3-(3,4-Dimethoxyphenyl)-2H-chromen-2-one* (**14**) Prepared as described in the general procedure from 2-(3,4-dimethoxyphenyl)acetic acid; yield 55%; mp 127–129 °C (MeOH); IR, ^1^H-NMR and ^13^C-NMR (CDCl_3_) as described in reference [45]. MS (EI) *m/z* 282 (M^+^, 100).

*3-(3,4,5-Trimethoxyphenyl)-2H-chromen-2-one* (**15**) was prepared using the general procedure from 2-(2,3,4-trimethoxyphenyl)acetic acid; 35% yield; mp 122–124 °C (MeOH); IR 2964, 2940, 1717, 1605; ^1^H-NMR (CDCl_3_) δ 7.80 (s, 1H), 7.55 (dd, *J* = 2.0, 8.0 Hz, 1H), 7.52 (dt, *J* = 2.0, 8.0 Hz, 1H), 7.35 (bd, *J* = 8.0 Hz, 1H), 7.30 (dt, *J* = 2.0, 8.0 Hz, 1H), 6.94 (s, 2H), 3.92 (s, 3H), 3.91 (s, 3H), 3.89 (s, 3H); ^13^C-NMR (CDCl_3_) δ 56.2, 56.2, 60.9, 106.0, 106.0, 116.4, 119.7, 124.5, 127.8, 128.1, 130.1, 131.4, 138.8, 139.5, 153.1, 153.3, 153.3, 160.5. MS (EI) *m/z* 312 (M^+^, 100).

*7-Hydroxy-3-phenyl-2H-chromen-2-one* (**16**) A solution of the 2,4-dihydroxybenzaldehyde (0.02 mol) and phenylacetyl chloride (0.04 mol) in dry acetone (200 mL) was refluxed with anhydrous K_2_CO_3_ (10 g) for 6 h on a water bath. Then, acetone was removed under reduced pressure and cold water (100 mL) was added to the mixture. The solid product was separated by filtration and washed with cold water (2 × 50 mL); yield 92%; mp 217–219 °C (CHCl_3_/MeOH); IR 3212, 1517, 1676, 1599; ^1^H-NMR as described in reference [38], ^13^C-NMR (CDCl_3_) 102.4, 112.0, 113.9, 123.1, 128.2 (5C), 129.1, 135.0, 141.1, 155.1, 161.6, 161.9. MS (EI) *m/z* 238 (M^+^, 72).

*8-Hydroxy-3-(4-methoxyphenyl)-2H-chromen-2-one* (**17**) was prepared from 2,3-dihydroxybenzaldehyde (0.02 mol) using the procedure described for **16**. Yield 90%; mp 223–225 °C (CHCl_3_/MeOH); IR 3392, 2994, 2935, 2835, 1901, 1697, 1604; ^1^H-NMR (MeOD) δ 7.83 (s, 1H), 7.63 (d, *J* = 8.7 Hz, 2H), 7.06 (br d, *J* = 7.6 Hz, 1H), 6.98 (dd, *J* = 1.4, 8.2 Hz, 1H), 6.94 (d, *J* = 8.7 Hz, 2H), 6.86 (dd, *J* = 1.4 Hz, 8.2 Hz, 1H), 3.82 (s, 3H); ^13^C-NMR (MeOD) δ 55.6, 55.6, 114.4, 114.4, 115.9, 120.4, 121.4, 125.8, 127.1, 128.3, 130.5, 130.5, 141.6, 144.3, 150.5, 160.8, 163.0. MS (EI) *m/z* 268 (M^+^, 100).

*4-Hydroxy-6-methoxy-3-phenyl-2H-chromen-2-one* (**18**) A solution of dimethyl 2-phenylmalonate (0.01 mol) and 4-methoxyphenol (0.01 mol) in diphenyl ether was heated for 3 h to 200–250 °C. When the liberation of methanol had stopped, the reaction mixture was heated in an oil bath at 300 °C for 2 h more. After cooling, the reaction mixture was diluted with toluene (10 mL), filtered off and washed with cyclohexane (20 mL). 91%; mp 215–217 °C (CHCl_3_/MeOH); IR 3337, 3085, 3061, 3028, 1659; ^1^H-NMR (MeOD) δ 7.43 (m, 2H), 7.43 (m, 3H), 7.42 (br s, 1H), 7.29 (d, *J* = 9.1 Hz, 1H), 7.17 (dd, *J* = 3.0, 9.1 Hz, 1H), 3.87 (s, 3H); ^13^C-NMR (MeOD) δ 55.4, 105.3, 106.0, 116.4, 117.2, 120.2, 128.1, 128.5, 128.5, 130.6, 130.6, 130.9, 147.0, 155.7, 160.7, 163.9. MS (EI) *m/z* 268 (M^+^, 82).

*4-Hydroxy-7-methoxy-3-phenyl-2H-chromen-2-one* (**19**) was prepared from 3-methoxyphenol (0.01 mol) using the same procedure described for **18**; yield 90%; mp 217–219 °C (CHCl_3_/MeOH); IR 3420, 3092, 1654, 1604, 1559; ^1^H-NMR (CDCl_3_) δ 7.78 (d, *J* = 8.7 Hz, 1H), 7.41 (m, 2H), 7.41 (m, 3H), 6.87 (dd, *J* = 2.1, 8.7 Hz, 1H), 6.81 (d, *J* = 2.1 Hz, 1H), 3.87 (s, 3H); ^13^C-NMR (CDCl_3_) 55.8, 100.4, 108.2, 112.5, 118.9, 124.8, 128.9, 129.7, 129.7, 130.4, 130.4, 137.5, 154.3, 159.8, 159.9, 163.4. MS (EI) *m/z* 268 (M^+^, 80).

*4-Chloro-7-methoxy-3-phenyl-2H-chromen-2-one* (**20**) Compound **19** (0.01 mol) and POCl_3_ (0.5 mL) was heated at 135 °C for 4 h, cooled to room temperature, and stored at 0 °C for 1 h. This mixture was decanted into ice cold and then extracted with EtOAc to afford **19**; yield 93%; mp 221–222 °C (MeOH); IR 3,065, 3,021, 1,703, 1,618, 1,590; ^1^H-NMR (CDCl_3_) δ 7.83 (d, *J* = 8.7 Hz, 1H), 7.43 (m, 2H), 7.43 (m, 3H), 6.93 (dd, *J* = 2.1, 8.7 Hz, 1H), 6.85 (d, *J* = 2.1 Hz, 1H), 3.90 (s, 3H); ^13^C-NMR (CDCl_3_) δ 55.9, 100.5, 112.2, 113.0, 123.8, 127.3, 128.3, 128.3, 128.7, 130.1, 130.1, 132.8, 146.0, 153.7, 160.0, 163.5. MS (EI) *m/z* 286 (M^+^, 94).

### 3.3. Anti-HIV Bioassay

The anti-HIV activity of these coumarins on Tat and NF-κB functions has been analysed. To this aim, we have used two stably transfected cell lines. The previously described 5.1 cell line [45] is a Jurkat-derived clone stably transfected with a plasmid containing the luciferase gene under the control of HIV-LTR. In this cell clone, activation with TNFα induces NF-*κ*B activation and subsequent HIV-1 expression. We have also analyzed the anti-HIV activity in HeLa-Tat-Luc cells, in which the HIV-1 LTR is directly activated by the HIV-1 Tat protein. A compound was considered active in one assay if it inhibited the target function by more than 50% (NF-*κ*B) or 30% (Tat) at either 25 or 50 μM concentration. The active compounds were submitted for further evaluation through a HeLa-Tet-ON assay, as previously described [41]. In the Hela-Tet-ON cells the luciferase expression is under control of an artificial promoter that can be activated by tetracycline. Therefore, compounds that inhibit tetracycline-induced luciferase activity were considered non-specific for luciferase-based anti-HIV assays and were not examined further, and those showing specific activity (non-inhibitors in HeLa-Tet-ON cells) were analysed using a recombinant virus (RV) assay. In addition, those compounds that were specific and did not reach the threshold of NF-*κ*B and anti-Tat activity were also tested in RV assay to rule out anti-HIV activity through other pathways. In this system, a luciferase reporter gene has been cloned in full-length infectious DNA. The measured luciferase activity is therefore directly proportional to the replication of the virus. This assay is more sensitive and reliable than classic infectious assays [43]. Cell viability was evaluated in non-infected treated cultures following the same protocol as in the recombinant virus assay and measuring cell toxicity with a classical MTT assay. IC_50_ and CC_50_ were calculated using GraphPad Prism software [non-linear regression, log (inhibitor) *vs.* response].

## 4. Conclusions

In summary, a small collection of simple 3-phenylcoumarins has been prepared and evaluated *in vitro* against two molecular targets of HIV-1 and also as viral replication inhibitors. Along with 4-phenylcoumarin skeleton, the 3-phenylcoumarin one can also be considered a scaffold for the design and development of new potential antivirals, acting through the specific inhibition of NF-*κ*B or the transcriptional Tat protein. Considering that simple changes in structure promote highly significant changes in bioactivity, further research should take into account these findings for the preparation of more potent and selective inhibitors of the NF-*κ*B and Tat viral pathways.
